# An Improved Model for Circular RNA Overexpression: Using the Actin Intron Reveals High Circularization Efficiency

**DOI:** 10.1002/ggn2.202200019

**Published:** 2022-10-09

**Authors:** Feiya Li, Juanjuan Lyu, Yang Yang, Qiwei Yang, Cristian Santos, Burton B. Yang

**Affiliations:** ^1^ Sunnybrook Research Institute Sunnybrook Health Sciences Centre Toronto ON M4N 3M5 Canada; ^2^ Department of Laboratory Medicine and Pathobiology University of Toronto Toronto ON M5S 1A8 Canada

**Keywords:** actin intron, circRNA, circular RNA, overexpression, splicing factor, T4 intron

## Abstract

Traditionally, the group 1 intron of the T4 *td* gene is used to generate a foreign circular sequence. However, the T4 system has been shown to be fairly inefficient in expressing circular RNA (circRNA). Here, a new method is developed to express circular sequences with high circularization efficiency to strengthen the confidence for future circRNA functional studies. CircRNA expression plasmids, constructed with different lengths derived from the actin intron (15‐nt, 30‐nt, 60‐nt, 100‐nt, 180‐nt) and T4 intron, are introduced into human and mouse cell lines 293T and B16. Junction detection and sequencing are used to determine successful circularization of introns and their expression efficiencies. An actin intron with a medium length (60‐nt–100‐nt) shows significantly increased efficiency of circularization, whereas intron‐100‐nt shows the best efficiency in most conditions. RNA pull‐down assays are designed to precipitate the splicing factors that are bound to the introns and intron/exon junction. The precipitated proteins are analyzed by mass spectrometry (MS), aiming to identify the possible underlying mechanism behind the high circularization efficiency. This expression system has been validated using different circRNAs, and such method shows potential in contributing to the expanding field of circRNA studies.

## Graphical abstract

It is essential to have high efficiency of circularization to study the functions of a circRNA. Constructs with different lengths of actin introns and T4 intron show that introns with medium lengths produce the best circularization efficiency. Pulling‐down circRNA co‐precipitates splicing factors that binds to the introns and intron/exon junction, which, after being revealed by mass spectrometry, allows mechanistic analysis.

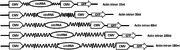

## 1 Introduction

Circular RNAs (circRNAs) are a type of noncoding RNAs that were discovered in mammals relatively recently in the 1990s,^[^ ^1^, ^2^, ^3^ ^]^ and have long been thought of as purposeless entities generated by aberrant RNA splicing. The majority of circRNAs are produced through back‐splicing of the linear precursor RNAs in which the 3’ splice site is covalently linked with the upstream 5’ splice site.^[^ ^3^, ^4^ ^]^ Additionally, circRNAs could result from 3’ exonucleolytic degradation of lariat RNAs, thus forming an intronic circRNA with a 2’‐5’ carbon linkage. The former, leads to the generation of an exon‐intron (EIciRNA) or exonic circRNA (EcircRNA), while the latter leads to the intronic circular RNA (ciRNA) generation.^[^ ^3^, ^5^, ^6^, ^7^, ^8^ ^]^ Although much research regarding circRNAs in the recent years aimed at increasing our understanding of these novel molecules, little is known about the structure‐function relationships they may have.

Many of the discovered circRNAs have unknown functions, however, they have been seen to be conserved across species and are expressed independently of their associated linear isoforms in a regulated fashion, indicating their potential physiological significance.^[^ ^9^, ^10^, ^11^ ^]^ Despite being relatively novel, circRNAs have been recognized for their advantageous features. For instance, the circular structure seen in circRNAs provides high stability due to exonuclease inaccessibility, as there are no free ends to be cleaved. Additionally, the circular structure has been shown to increase the median half‐life to over 48 h compared to the average half‐life of 10 h for linear mRNAs.^[^ ^3^ ^]^ The circular structure can also act as a template for rolling protein synthesis, as has been shown with engineered circRNAs both in vitro and in vivo.^[^ ^11^, ^12^ ^]^ Although there are some endogenous circRNAs that display translation capabilities, most of these are incapable of translation as they do not seem to be associated with ribosomes.^[^ ^11^ ^]^ However, increasing studies have made efforts to unveil different modes of circRNA translation and the related functions.^[^ ^13^ ^]^ Moreover, circRNAs have been shown to act as microRNA sponges by causing inhibitory or excitatory effect on miRNA expression, mediated by multiple miRNA binding sites within their circular sequence.^[^ ^14^ ^]^ Furthermore, circRNAs interact with the circRNA binding proteins (cRBPs) and regulate their functions.^[^ ^15^, ^16^, ^17^ ^]^

As many studies are emerging concerning the functions of specific circRNAs, a quality platform must be built to combat the current limitation of low circRNA expression. When conducting a functional study, the most common technique is to transfect circRNA expression constructs into the cells, which is then compared with the cells transfected with control vectors.^[^ ^5^, ^18^, ^19^, ^20^, ^21^, ^22^, ^23^, ^24^ ^]^ The difference in expression between the two groups of cells displays the functions of the circRNA in question. The traditional way to introduce a circRNA plasmid is to integrate the group 1 intron of the phage T4 *td* gene into the expression construct, where a foreign sequence is introduced with this intron for circularization.^[^ ^25^ ^]^ However, this system has brought about some uncertainties in various functional studies owing to low efficiency of circularization, and is not ideal for circRNA overexpression.^[^ ^26^, ^27^ ^]^ For this reason, a new method to efficiently generate circRNAs endogenously is highly needed.

It has been elucidated that the intron, through its importance in circRNA biogenesis, is the main factor when it comes to the construction of an expression construct that can successfully generate the circRNA accurately and efficiently.^[^ ^3^, ^4^, ^28^ ^]^ With this in mind, we have selected the intron from human beta‐actin gene that undergoes constitutive splicing, rather than alternative splicing. The advantages of using an actin intron include: i) actin is a housekeeping gene that is widely expressed throughout the cells and tissues. Since all types of cells express the necessary splicing factors, it has potential to overexpress circRNA of interest in any cell type; ii) using an actin intron will not potentially affect the function of any circRNA of interest, further ensuring the specificity when investigating circRNA function; iii) while introns from other genes have alternative splicing, the intron we used does not, making it more controllable in an expression system. By comparing with the traditional phage T4 *td* gene system, varying lengths of the actin intron were used to determine the optimal form. Our results revealed that the 100‐nt actin intron length is the optimal length for endogenous circRNA expression. We further identified the specific splicing factors that are bound to actin intron 100, which provided a possible explanation for its high circularization efficiency. This actin intron overexpression system has been further validated with more circRNA candidates and has successfully aided in characterizing circRNAs functions.^[^ ^16^, ^18^, ^19^, ^20^, ^21^, ^22^ ^]^

## 2 Results

### 2.1 Actin Intron 100 Shows the Highest circRNA Circularization Efficiency

**Figure 1:**
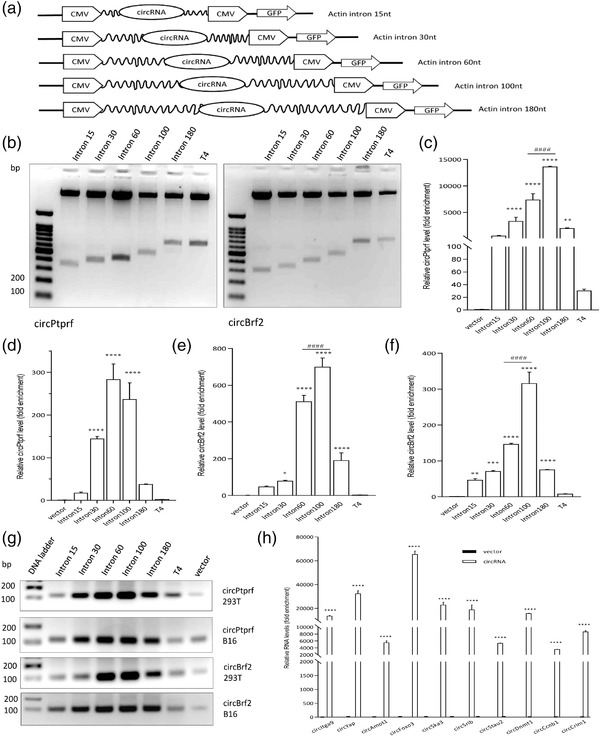
Circularization efficiency affected by different sizes of introns. a) Structures of expression constructs. b) Left, Agarose gel electrophoresis showing different sizes of introns constructed into the circPtprf plasmid. Right, Agarose gel electrophoresis showing different sizes of introns constructed into the circBrf2 plasmid. From left to right, the wells include: 100 bp DNA ladder, intron 15, intron 30, intron 60, intron 100, intron 180, and T4. The bands showed the different sizes of DNA fragments between two CMV promoters in Panel (A). c) 293T cells were transfected with circPtprf with introns of different types and sizes as shown. Circularization efficiency was tested by RT‐qPCR. The actin intron of 100 nucleotides displayed the best circularization efficiency by producing the highest amounts of PCR products. **p < 0.01, ****p < 0.0001, One‐way ANOVA, compared to vector. ###*p* < 0.001, One‐way ANOVA, compared to intron 60. d) B16 cells were transfected with circPtprf with introns of different types and sizes as shown. Circularization efficiency was tested by RT‐qPCR. Actin intron 60 shown highest circularization efficiency. ****p < 0.0001, One‐way ANOVA, compared to vector. e) 293T cells were transfected with circBrf2, followed by testing the circularization efficiency. Actin intron 100 showed the highest circularization efficiency. *p < 0.05, **** *p* <0.0001, One‐way ANOVA, compared to vector. ###*p* < 0.001, One‐way ANOVA, compared to intron 60. f) B16 cells were transfected with circBrf2, followed by testing the circularization efficiency. Actin intron 100 produced the best results. **p < 0.01, ***p < 0.001, ****p < 0.0001, One‐way ANOVA, compared to vector. ###*p* < 0.001, One‐way ANOVA, compared to intron 60. g) 293T and B16 cells were transfected with circPtprf or circBrf2 with introns of different types and sizes as shown. Circularization efficiency was tested by RT‐PCR, followed by agarose gel electrophoresis. The actin intron of 100 nucleotides displayed the best circularization efficiency by producing the highest amounts of PCR products. h) Different circRNAs were inserted and expressed using intron 100 plasmid including circItga9 (316 bp), circYAP (296 bp), circAMOTL1 (922 bp), circFOXO3 (1435 bp), circSKA3 (412 bp), circSCRIB (138 bp), circStau2 (296 bp), circDNMT1 (155 bp), circCCNB1 (342 bp), and circCRIM1 (364 bp). Each plasmid was transfected with vector control into 293T cells for at least 6 h. Samples were collected for RNA extraction and RT‐qPCR using specific junction detection primers. All circRNAs overexpressed at a high fold enrichment compared to the vector control. ****p < 0.0001, Student t‐test within each circRNA group.

Two circRNAs, circPtprf (270 bp) and circBrf2 (322 bp), were chosen as inserts and constructed into the plasmids with different sizes of an actin intron (15‐nt, 30‐nt, 60‐nt, 100‐nt, and 180‐nt) or phage T4 *td* intron (**Figure** **1**a; Table S1, Supporting Information). The green fluorescent protein (GFP) driven by the human cytomegalovirus (CMV) immediate‐early enhancer and promoter was engineered into the plasmid for convenient visualization of the successful delivery of the plasmids. The correct size of each intron was detected through PCR and the bands with expected sizes were resolved through 2% agarose gel electrophoresis analysis. As shown in Figure 1b, every intron was confirmed to be correctly expressed with their expected sizes, demonstrated by their positions relative to the 100‐bp DNA ladder on the left side of the images.

Next, we examined the intron which expressed the circRNAs at the highest efficiency. To investigate the system in a more universal manner, we transfected the circRNA plasmids with different introns into human 293T cells and mouse B16 cells. We designed primers that specifically targeted circPtprf and circBrf2, respectively. Successful circRNA production was monitored by reverse transcription and quantitative polymerase chain reaction (RT‐qPCR). We found that comparing to the T4 intron, all actin introns showed significantly higher activities in facilitating circRNA expression (Figure 1c–f). While all the actin introns facilitated successful circRNAs expression, the actin intron 60 and actin intron 100 showed substantially higher efficiency in producing circRNAs relative to the controls, whereby the intron 100 had the highest activity in most cases.

Having resolved the PCR products in the gel visualized under UV, it also showed that T4 intron, even though it successfully generated the circRNAs, expressed the circRNAs at the lowest level compared with the other actin introns (Figure 1g). Among the different lengths of the actin introns, intron 100 showed the highest circularization efficiency.

To validate the system in a more universal manner, more circRNAs were tested using the actin intron 100 overexpression model. As shown in Figure 1h, 10 circRNAs with different sizes were constructed using the plasmid with actin intron 100. Following overexpression of each circRNA plasmid in the cells, specific primers detecting each circRNA junction were used for RT‐qPCR. Compared with the vector control, the circRNA actin intron 100 plasmid showed magnificently increased levels of circRNAs. The results suggested that this actin intron 100 system is not only circularizing the chosen candidate circPtprf and circBrf2 correctly, but also displaying high efficiency for expression of these circRNAs in a universal manner. This overexpression system showed its potential in being adaptable to express any circRNA with different sizes for the functional studies.

**Figure 2:**
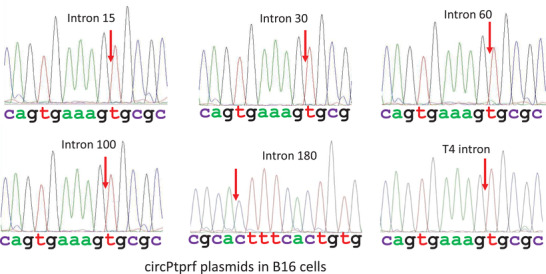
“Head‐to‐tail” junctions of the constructs. B16 cells were transfected with circPtprf plasmids with different introns. Collected samples were subjected to PCR and resolved on gel. The gels were cut and purified for junction sequencing. Red arrows: “head‐to‐tail” junction points.

To verify the correct splicing and circularization, circPtprf and circBrf2 expressed in murine B16 cells were amplified by RT‐PCR with divergent primers and subjected to Sanger sequencing. By using the murine cells in the analysis, we excluded the potential interference of the endogenous human circPtprf and circBrf2 for the Sanger sequencing. The predicted “head‐to‐tail” junction was confirmed for every intron plasmid. The sequencing results showed successful circularization of the exogenous circPtprf (**Figure** **2**) and circBrf2 (Figure S1, Supporting Information) expression using different intron plasmids in the cells. The results also suggested that T4 intron was able to generate correct circRNAs, but with lower efficiency. Different lengths of the actin introns were all able to correctly express exogenous circRNAs, but with different efficiencies.

**Figure 3:**
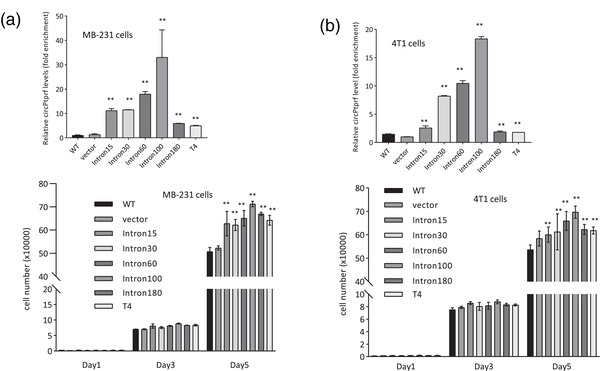
Functional assays of the constructs. a) Upper, human breast cancer cell line MDA‐MB‐231 was transfected with different constructs of circPtprf generated using different intron sets as shown. Significantly higher levels of circPtprf circularization were detected. Lower, in cell proliferation assay on day 5, increase in cell proliferation was observed with these circPtprf constructs. **p < 0.01, One‐way ANOVA, compared to vector. b) Upper, mouse breast cancer cell line 4T1 was transfected with different constructs of circPtprf generated using different intron sets as shown. Significantly higher levels of circPtprf circularization were detected. Lower, in proliferation assay on day 5, increase in cell proliferation was observed with these circPtprf constructs. **p < 0.01, One‐way ANOVA, compared to vector.

To test whether enhanced circularization had any biological function, we performed some functional assays. All circPtprf expression constructs generated by different intron sets were transfected into human breast cancer cell line MDA‐MB‐231 (**Figure** **3**a) and mouse breast cancer cell line 4T1 (Figure 3b). After confirming enhanced expression of these constructs (Figure 3a,b, upper), we detected increased proliferation rates of the cells transfected with these constructs (Figure 3a,b, lower). All circPtprf expression constructs were subjected to the same assays and similar patterns of expression were observed (Figure S2a,b, Supporting Information). In cell proliferation assays, expression of circPtprf constructs resulted in decreased cell proliferation (Figure S2c,d, Supporting Information).

### 2.2 Effects of Splicing Factors on Circularization Efficiency

**Figure 4:**
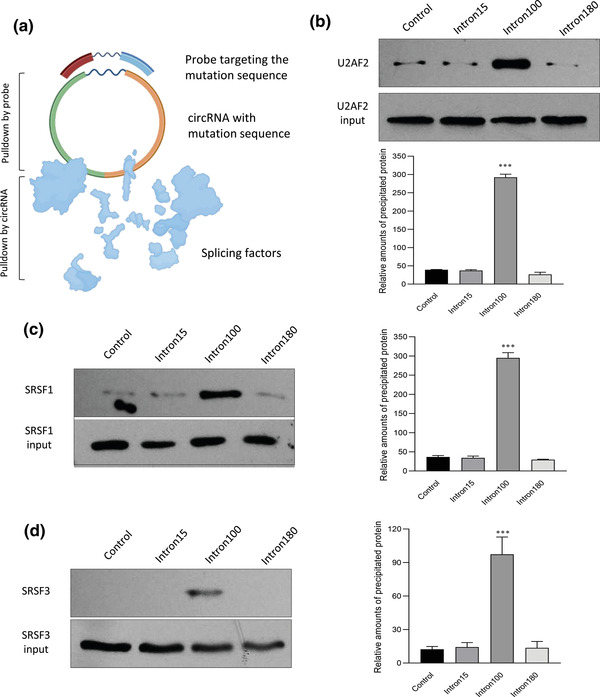
Pull‐down assay confirming the binding between actin intron 100 and the splicing factors. a) A probe was designed to bind to the mutation sequences of each construct. b–d) 293T cells were transfected with circPtprf intron plasmids (intron 15 as control, mut‐intron 15, mut‐intron 100, mut‐intron 180) and subjected to the pull‐down assay using probe targeting the mutated fragment. Intron 15 served as a control, while other mutant plasmids were shown as intron length. Western blot representatives and intensity analysis showing that the probe targeting mut‐intron 100 pulled down significantly higher levels of b) U2AF2, c) SRSF1, and d) SRSF3 compared with probes targeting other mutant constructs. Each pull‐down experiment was repeated for at least 3 times. ***p < 0.001 versus control.

**Figure 5:**
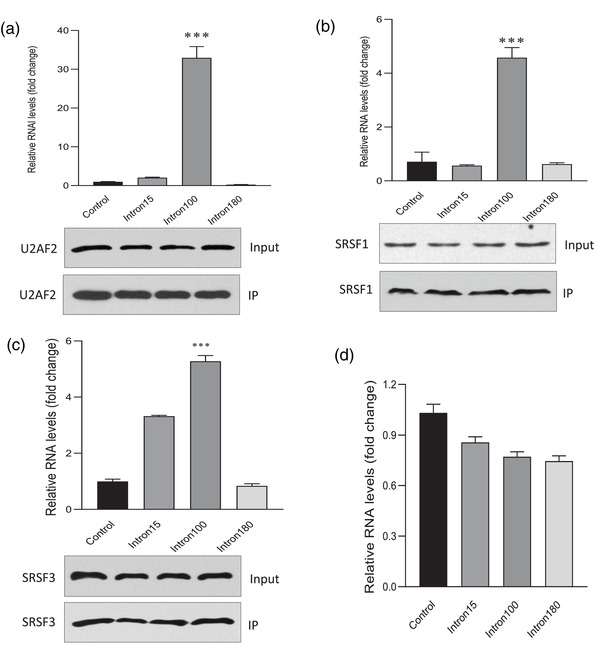
Immunoprecipitation showing the binding between actin intron 100 and the splicing factors 293T cells were transfected with circPtprf intron plasmids (intron 15, mut‐intron 15, mut‐intron 100, mut‐intron 180). Intron 15 served as a control, while other mutant plasmids were shown as intron length. Antibodies against A) U2AF2, B) SRSF1, and C) SRSF3 precipitated significantly higher levels of mut‐intron 100 relative to the other mutant constructs. Successful precipitation of the proteins by each antibody was provided in the figure (Input, protein levels in each sample; IP, proteins immune‐precipitated by the antibodies in each sample). D) The amounts of RNAs in each input group showed little difference. Each precipitation experiment was repeated for 3 times. ***p < 0.001 versus control.

To uncover the underlying mechanisms behind increased circularization efficiency and expression of the actin intron 100, we performed a circRNA pull‐down assay and subjected the products to LC‐MS/MS. While the circRNA pull‐down assay is a powerful method to detect potential binding partners of the circRNA, the splicing factors that potentially bind to the introns facilcircularization facilitating should be detected. To identify these splicing factors, we modified the circRNA pull‐down assay by constructing a plasmid with a mutated sequence in the middle of circPtprf and generated a probe that specifically targeted that mutant sequence (**Figure** **4**a). The mutation sequences were designed in the center of the circRNAs. It was expected not to affect the circularization specificity and efficiency. Since the probe was 100% complementary to the mutant sequence, it would only pull down the mutant intermediates but not the endogenous circPTPRF nor its mRNA counterpart (linear PTPRF mRNA). By doing this, instead of detecting potential binding partners of the circRNA inserts, we were able to pull down the splicing factors that bind to these specific introns introduced into the system specifically during circularization. The proteins precipitated by circRNA pull‐down assay were sent for mass spectrometry analysis. The constructs included mutated intron 15, mutated intron 100, mutated intron 180, and non‐mutated intron 15 (negative control). The precipitated splicing factors were identified and shown in **Table** **1**, where intron 100 showed significantly higher binding affinity to the splicing factors, U2AF2, SRSF1, and SRSF3. To confirm the mass spectrometry results and test the specificity of the interaction, we performed circRNA pull‐down assay followed by western blot using antibodies against U2AF2, SRSF1, and SRSF3, respectively. As shown, the probe targeting mut‐intron 100 pulled down more U2AF2 (Figure 4b), SRSF1 (Figure 4c), and SRSF3 (Figure 4d) compared to the other actin intron lengths and the negative control. To double confirm the binding results, the antibodies against these specific splicing factors were subjected to immunoprecipitation analysis. Successful precipitation of the proteins by the antibodies used was confirmed by Western blotting where similar results were obtained (**Figure** **5**). Antibodies against U2AF2, SRSF1, and SRSF3 precipitated significantly higher levels of mut‐intron 100 relative to the others indicating specific binding between intron 100 and U2AF2, SRSF1, and SRSF3 (Figure 5). The binding of splicing factors to intron 100 provided a potential explanation underlying the high circularization efficiency of this actin intron length.

**Table 1:** Mass spectrophotometry results showing splicing factors binding to introns

Accession Number	Alternate ID	Molecular Weight	intron 15	mut‐intron 15	mut‐intron 100	mut‐tintron 180	
P23246 (+1)	SFPQ	76 kDa	7	10	10	4	
P26368 (+1)	U2AF2	54 kDa	3	2	10	2	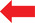
Q07955 (+2)	SRSF1	28 kDa	3	3	10	3	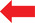
Q6P2Q9	PRPF8	274 kDa	5	6	5	2	
O43143	DHX15	91 kDa	4	5	3	3	
P84103 (+1)	SRSF3	19 kDa	2	4	7	2	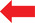
Q9UHX1‐2	PUF60	58 kDa			2	2	
Q13435	SF3B2	100 kDa	3	1	3	2	
Q13243	SRSF5	31 kDa	2	2	4	3	
O75494 (+5)	SRSF10	31 kDa	3	5	3		
Q15393	SF3B3	136 kDa	4	2	1	1	
O75533	SF3B1	146 kDa	2	1	3	2	
Q16629 (+3)	SRSF7	27 kDa	3	4	6	2	
Q15427	SF3B4	44 kDa	2	2	1	1	
Q01130 (+1)	SRSF2	25 kDa	1		3		
Q08170 (+2)	SRSF4	57 kDa				2	
Q12874	SF3A3	59 kDa	1			1	
P0DN76 (+3)	U2AF1L5	28 kDa			1		
Q13242	SRSF9	26 kDa			1		

Arrows show splicing factors potentially interacting with Actin intron 100.

**Figure 6:**
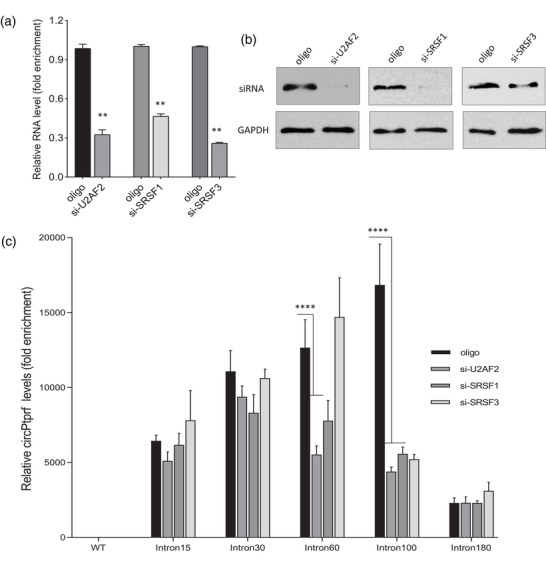
Silencing splicing factors decreased the circularization efficiency. The circPtprf constructs containing different intron lengths were co‐transfected with siRNAs targeting U2AF2, SRSF1, and SRSF3 in 293T cells. Silencing these molecules were confirmed by a) real‐time PCR and b) Western blotting. **p < 0.01, One‐way ANOVA comparing with oligo control of each group. c) While intron 100 showed the highest circularization efficiency when being co‐transfected with the control oligo (si‐Oligo), this effect was inhibited in co‐transfection with siRNAs targeting U2AF2, SRSF1, and SRSF3. Intron 60 possessed the second highest circularization efficiency, and this effect was blocked by co‐transfection with si‐U2AF2 and si‐SRSF1. ***p < 0.001, One‐way ANOVA comparing with oligo control of each group.

To further confirm the regulatory effects of the specific splicing factors on actin introns, we co‐transfected different circPtprf intron plasmids with siRNAs targeting each splicing factor or a control oligo. All siRNAs were confirmed to silence U2AF2, SRSF1, and SRSF3 in 293T cells by real‐time PCR (**Figure** **6**a) and Western blotting (Figure 6b). We found that in the control oligo group, circPtprf containing intron 100 expressed the highest levels of circularized products, but this effect was abolished after co‐transfection with U2AF2 siRNA, SRSF1 siRNA, or SRSF3 siRNA (Figure 6c; Table S2, Supporting Information).

## 3 Discussion

In this study, we explored the novel and prolific field of circRNAs and their overexpression system for the functional studies. An efficient system for circRNA overexpression, using the actin intron 100, was designed and characterized. Compared to the traditional T4 system, the actin introns facilitate the generation of circRNAs with correct junctions precisely and efficiently, among which the actin intron 100 showed the highest efficiency in circRNAs generation. Through RNA pull‐down, followed by a mass spectrometry assay, actin intron 100 demonstrated significantly increased binding affinity to three splicing factors, U2AF2, SRSF1, and SRSF3. Further immunoprecipitation analysis along with the siRNA treatments confirmed that the high efficiency found in the intron 100 could be explained by the binding of specific splicing factors to the intron. Altogether, these results have demonstrated that the actin intron 100 has greater circularization efficiency when it comes to introducing foreign sequences in the transfected cells. This is a major discovery in the field of circRNA as it has introduced an improved way to overexpress circRNAs in the cells of choice and later could be used in the animal models. Discovering an improved model will bring about more discoveries with higher confidence, and it may be worth reproducing previous studies that used the T4 *td* gene system, since that system has now been proven to be inefficient.

The use of actin introns for circRNA generation in the transfected cells has rarely been reported in the literature. Other than T4 *td* intron system, the minimal ZKSCAN1 introns with the EcoRV and SacII sites is another expression system that has been validated for circRNA generation.^[^ ^29^ ^]^ In this study, we chose actin intron for building up a new expression system and aimed at adapting the system to a universal use. This actin intron system is very effective in introducing the circRNA constructs into transfected cells, demonstrating much higher efficiency than the T4 *td* gene system. The results from the MS assay showed that the actin intron 100 has significantly higher efficiency of binding to the splicing factors U2AF2, SRSF1, and SRSF3. The U2AF2 splicing factor has been shown to bind directly upstream of the 3’ splice site at a uridine/cytidine‐rich sequence element, and it is essential in recruiting a subunit of the spliceosome, the U2 subunit.^[^ ^30^ ^]^ SRSF1 has also been known as a regulator of RNA splicing, in that, it promotes the use of alternative 5’ and 3’ splice sites.^[^ ^31^, ^32^ ^]^ Together with U2AF2, SRSF1 plays an important role in definition of the 3’ splice site. As well, SRSF3 regulates many splicing variants, oftentimes through the selection of an alternative 3’ splice site.^[^ ^33^ ^]^ In our study, the preferential binding of these factors to actin intron 100 helps explain the reason for its increased expression efficiency. These factors aid in the excision of the intron from the plasmid, mainly at the 3’ end, which then covalently links to the 5’ end and forms a circRNA.

While smaller introns are easier to be spliced out by the splicing machinery, an intron with a sufficient length ensures the accuracy of generating a circRNA and provides more binding sites for the splicing factors. However, this is not a general rule for all the large introns. As shown in this study, intron 180 did not generate sufficiently good results in terms of circularization efficiency. An interesting finding here is the specific binding of SRSF4 to the intron 180 (Table S3, Supporting Information). This may be an attributing factor to play role in its reduced efficiency of circularization and expression as seen with intron 180. Too long of an intron may allow for increased binding of the inhibitory factors, therefore, inhibiting either the excision of the intron from the plasmid or its circularization. As well, the binding activities of proteins to RNAs depend on their affinities. If some splicing factors have higher affinities to the RNA fragment containing 180 nucleotides than the other splicing factors binding to the RNA fragment containing the 100 nucleotides, it would be possible that in the presence of a longer nucleotide fragment, the interaction of the splicing factors with the 100‐nucleotide fragment decreased. Another possibility is spatial obstacle that the 180‐neucleotide fragment inhibited the interaction of the splicing factors with the 100‐nucleotide fragment. These await further investigation.

## 4 Experimental Section

### Plasmid Generation

Plasmids were generated by ligating a vector with the target insert. Refer to Table S4 (Supporting Information) for full information on plasmid sequences. The plasmid vector was generated by digesting 40 µL plasmid YAP by BamHI and XhoI (NEW ENGLAND BIOLABS) at 37 °C for 2 h. This product was digested with HindIII and SalI (NEW ENGLAND BIOLABS) at 37 °C for 2 h, and then incubated at 70 °C for 10 min to inactivate the enzymes. 15 µL sodium acetate (3 mol L^−1^, pH 5.2) and 330 µL pre‐cool 100% ethanol at −20 °C were added to the mixture for 30 min to precipitate the DNA. The DNA was precipitated by centrifugation at 4 °C for 30 min. The supernatant was removed, and the pellet was air dried and resuspended in 50 µL TE buffer to get the vector. The target DNA inserts were obtained by three rounds of PCRs. Detailed information of primers could be found in Table S5 (Supporting Information). First, primers 32.51/52 and 32.53/54 were used in 15 cycles PCRs for amplifying Ptprf and Brf2, respectively, with the annealing temperate of 56 °C. The PCR products were diluted in distilled H_2_O (100 times). The diluted PCR products in this step were saved as a template for the next step. Next, primers 32.19/20 and primers 32.17/18 were used in 15 cycles PCRs for amplifying the actin intron and T4 intron, respectively with the annealing temperate of 56 °C. The PCR products were diluted 100 times in distilled H_2_O. The diluted PCR products in this step were saved as a template for the next step. Finally, Primers 32.47/48 and primers 32.45/.46 were used in 30 cycles PCRs for amplifying the intron 180 and T4 intron, respectively with the annealing temperate 56 °C. The products from the last step were subjected to gel electrophoresis containing 2% agarose which was then purified (refer to “Gel Purification”) to get the insert for ligation. The vector and insert were ligated at 16 °C overnight with T4 DNA ligase (NEW ENGLAND BIOLABS) to get Ptprf and Brf2 intron 180 and intron T4. Different intron primers, 32.67/68 (intron 15), 32.69/70 (intron 30), 32.71/72 (intron 60), and 32.49/50 (intron 100) were used to perform PCR using the intron‐180 plasmid as a template to get circPtprf and circBrf2 intron 15, 30, 60 and 100 inserts. The different intron inserts were ligated with the vector to get circPtprf and circBrf2 intron 15, 30, 60, and 100 plasmids. The original data are provided in figShare.^[^ ^34^ ^]^

### Agarose Gel Electrophoresis, DNA Purification, and Plasmid Amplification

Two percent agarose gel was prepared for electrophoresis. 20 µL DNAs were loaded into each well mixed with 6X loading dye (NEW ENGLAND BIOLABS). Electrophoresis was performed at 100 V for 30 min. 100 bp DNA ladder (FroggaBio) was used as size marker. Once the electrophoresis was stopped, the gel was analyzed under UV and pictures were taken with SYNGENE software.

After gel electrophoresis, the desired DNA fragments were visualized against UV light and the fragments were selected after comparing against a molecular weight standard. The gel was cut with a razor blade. After cutting the desired DNA, the gel was placed in a microfuge tube at −80 °C for 30 min. The gel was squeezed to collect the liquid and 1:1 phenol/chloroform was added. The mixture was then vortexed, followed by centrifugation at 12 000–16 000 xg for 2 min, and the colorless upper aqueous phase was transferred to a fresh tube (≈200 µL). 20 µL 3 mol L^−1^ NaAC and 500 µL 100% ethanol were added, and the mixture was shaken vigorously by vortex. The mixture was then placed at −80 °C overnight, followed by centrifugation at 12 000–16 000 xg at 4 °C for 30 min. The supernatant was removed, and the pellet was dried. The DNA was then dissolved in 20 µL distilled H_2_O.

The DNA fragments containing the circRNA sequence and the 5’ and 3’ introns were ligated with the expression vector assisted by T4 DNA ligase following the standard procedure. The ligation mix (10 µL) was gently mixed with 50 µL of bacteria TOP10 chemically competent *E. coli* (Invitrogen). The mixture was incubated on ice for 30 min, followed by incubation at 42 °C for 30 s and incubation on ice for 2 min. 250 µL of pre‐warmed SOC medium was added, and the culture was placed in a 37 °C shaking incubator slowly moving at 225 rpm for 1 h. 200 µL of the mixture from each incubation tube was evenly spread onto a pre‐warmed agar plate containing the antibiotic. The plates were upside down and incubated at 37 °C overnight. On day 2, individual clones were selected and cultured in LB medium in a 37 °C shaking incubator overnight. On day 3, a mini plasmid kit (Geneaid) was used to extract the plasmids. The products were then digested with BamHI and XhoI, and separated in 2% agarose gel. The insert size was compared with a molecular weight standard to identify the clone containing the candidate insert.

### Circular RNA Expression and Isolation

Human 293T cells were cultured in 6‐well tissue culture plates and transfected with 0.2 µg DNA plasmids dissolved in 10 µL serum‐free DMEM and mixed with 2.0 µL Polyjet. The mixture was incubated at room temperature for 15 min. At this point, the culture medium was removed, and the cells were rinsed with serum‐free DMEM. Then, 1 mL serum‐free DMEM was added to the rinsed cultures per well. The DNA‐Polyjet mixture was slowly added to the cultures. The cultures were maintained at 37 °C for 4–6 h allowing the DNA to be taken‐up by the cells. The serum‐free medium was then replaced with the medium containing 10% FBS. The cells were harvested by treating them with trypsin‐EDTA two days after the transfection and kept at −80 °C in a freezer or used for RNA isolation.

Total RNA was extracted with the Geneaid TriRNA pure kit. 80–100% confluent cells were harvested from the 6‐well plates after being washed with PBS twice. 600 µL GENEzol Reagent was added to the well and left for 3 min. The lysate was transferred to a 1.5 mL tube, followed by centrifugation at 12 000–16 000 xg for 1 min to remove cell debris. The supernatant was transferred to a microcentrifuge tube (RNase‐free). One volume of absolute ethanol was added directly to one volume of sample mixture (1:1) in the GENEzol Reagent. The sample mixture was transferred to the RB Column and centrifuged at 14 000–16 000 xg for 1 min. The flow‐through was discarded. 400 µL Pre‐Wash Buffer was added to the RB column, followed by centrifugation at 14 000–16 000 xg for 30 s. 600 µL Wash Buffer was added to the RB column, followed by centrifugation at 14 000–16 000 xg for 30 s. This step was repeated once. The RB columns were subjected to centrifugation at 14–16 000 xg for 3 min to remove any trace amount of the liquid left, and were placed into a new 1.5 mL tube. 30 µL of RNase‐free water was added to the center of the column matrix, followed by incubation at room temperature for at least 3 min to have maximum elution of the products. This was performed by centrifugation at 14–16000 xg for 1 min to collect the purified RNA.

### Real‐Time PCR

cDNAs were synthesized by reverse transcription for further use. Before reverse transcription, the purified RNA concentrations were measured using Nanodrop2000. In a 20 µL system, 1 µg RNA was used as a reverse transcription template, 4 µL of iScript Reverse Transcription Supermix was added, and nuclease‐free water was added to reach the final volume. The complete reaction mixture was incubated at 25 °C for 5 min, at 46 °C for 20 min, and at 95 °C for 1 min to synthesize the cDNA. cDNAs were placed at 4 °C for immediate use. For longer periods of storage, they could be kept at −20 °C. Real‐time PCRs were performed with SsoAdvanced Universal SYBR Green Supermix Kit (BIO‐RAD), using 1 µL cDNA as a template. The primers used as real‐time PCR controls were Hu‐U6 RNA forward and Hu‐U6 RNA reverse.

RT‐PCR was performed using the cDNA as a template and the primers 31.71/31.72 and 31.73/31.74 for Ptprf and Brf2, respectively. The PCR products were subjected to electrophoresis in 2% agarose for 30 min. The gel was cut and frozen at −80 °C overnight. DNA in the gel was purified and resuspended in 30 µL TE buffer. A TA Cloning Kit (Invitrogen) was used for cloning the isolated DNA. 4 µL purified DNA was mixed with 1 µL salt solution and 1 µL vector gently and incubated at room temperature for 30 min. The reaction mixture was placed on ice, and the plasmid amplification occurred as above. Bacterial clones were selected and grown in LB at 37 °C overnight. The plasmids were extracted using the Mini Plasmid kit (Geneaid). To confirm that the junction was correct, the M13 Forward and M13 Reverse primers were used for DNA sequencing. The original data are provided in figShare.^[^ ^34^ ^]^

### Functional Assays

Human breast cancer cell line MDA‐MB‐231 and mouse breast cancer cell line 4T1 were cultured in complete medium (10% FBS and 1% antibiotic). The cells were transfected with the plasmids circPtprf and circBrf2 with different introns (vector control, intron 15, intron 30, intron 60, intron 100, intron 180, intron T4) one day before seeding. Transfected cells were seeded in 12‐well culture plates at 1 × 10^4^ – 2 × 10^4^ cells per well and cultured at 37 °C. The cells were collected, and the numbers were counted at Day 1, 3, and 5 using Cytation 5 cell image multi‐mode reader (BioTek Instrument) with bright field.

### siRNA Transfection

siRNA was resuspended using 150 uL DEPC H_2_O to make 1 OD solution (20 µM). Cells were seeded in 6‐well plates and transfected with the siRNAs at 15 µL mL^−1^ using Polyjet. Cells were collected 24 h after transfection and subjected to further applications (RNA extraction or western blot).

### Immunoprecipitation Assays

Immunoprecipitation assays were performed using magnetic beads (SureBeads, Cat#161‐4013, Bio‐Rad). Briefly, 100 µl magnetic beads were washed in PBS‐T (PBS + 0.1% Tween 20) and incubated with 5 µg primary antibody (U2AF2, Cat# A4552, ABclonal; SRSF1, Cat# A1649, ABclonal; SRSF3 Cat# A9054, ABclonal) at room temperature for 30 min. Cells were lysed with the CO‐IP buffer and incubated with antibody‐containing beads at room temperature for 1 h. The magnetic beads were washed 3 times with PBS‐T and either resuspended in Trizol for RNA extraction or resuspended in 2 × Laemmli buffer (0.125 M Tris‐HCl, 4% SDS, 20% glycerol, 10% 2‐mercaptoethanol, 0.004% bromophenol blue, pH 6.8) for western blot analysis.

### Western Blot

Cells were lysed and subjected to sodium dodecyl sulfate‐polyacrylamide gel electrophoresis (SDS‐PAGE) containing 5–15% acrylamide. Nitrocellulose membrane was used for protein transfer in 1x Tris/glycine buffer containing 20% methanol at 15 V for 1 h. The membrane was blocked in 5% non‐fat dry milk prepared in TBST buffer (10 mm Tris‐Cl, pH 8.0, 150 mm NaCl, 0.05% Tween‐20) at room temperature for 1 h and then incubated with primary antibodies (U2AF2, Cat# A4552, ABclonal; SRSF1, Cat# A1649, ABclonal; SRSF3 Cat# A9054, ABclonal) at 4 °C overnight. Next day, the membranes were washed with TBST buffer three times (10 min each) and incubated with secondary antibodies at room temperature for 1 h. The membranes were washed three times (20 min each), followed by visualization using ECL detection kit (Cat# C72652, Millipore Sigma). The original data are provided in figShare.^[^ ^34^ ^]^

### RNA Pull‐Down Assays

Using intron 15, 60, 180 plasmids with mutation as samples and intron 15 without mutation as a control, we precipitated the pre‐transcripts with a probe binding to the mutation area. In brief, 293T cells were cultured and transfected as described above in 100 mm culture plates. Detailed pull‐down process and preparation steps are provided in Chapter 2.2 Protocol for specific and efficient pulldown circRNA‐binding proteins. In this study, probe sequence was designed against the mutated sequence. A biotinylated circRNA probe was pre‐treated at 95 °C for 10 min and immediately placed in ice‐containing icing water (0 °C) for 5 min and then incubated with prepared lysates at room temperature for 2 h followed by another 1 h incubation with streptavidin magnetic beads (Invitrogen). Beads were washed briefly with the Co‐IP buffer for at least 6 times. 20 µL distilled H_2_O was added to the beads, and the mixture was incubated at 65 °C for 10 min for eluting the bound materials. The bound products were analyzed by LC‐MS/MS.

## Conflict of Interest

The authors declare no conflict of interest.

## Supporting information

Supporting Information

## Data Availability

The data that support the findings of this study are available from the corresponding author upon reasonable request.

## References

[1] H. L. Sanger , G. Klotz , D. Riesner , H. J. Gross , A. K. Kleinschmidt , Proc. Natl. Acad. Sci. USA 1976, 73, 3852. 1069269 10.1073/pnas.73.11.3852 PMC431239

[2] J. M. Nigro , K. R. Cho , E. R. Fearon , S. E. Kern , J. M. Ruppert , J. D. Oliner , K. W. Kinzler , B. Vogelstein , Cell 1991, 64, 607. 1991322 10.1016/0092-8674(91)90244-s

[3] W. R. Jeck , N. E. Sharpless , Nat. Biotechnol. 2014, 32, 453. 24811520 10.1038/nbt.2890 PMC4121655

[4] Y. Zhang , X.‐O.u Zhang , T. Chen , J.‐F. Xiang , Q.‐F. Yin , Y.u‐H. Xing , S. Zhu , L.i Yang , L.‐L. Chen , Mol. Cell 2013, 51, 792. 24035497 10.1016/j.molcel.2013.08.017

[5] F. Li , Q. Yang , A. T. He , B. B. Yang , Semin. Cancer Biol. 2021, 75, 49. 33035655 10.1016/j.semcancer.2020.10.002

[6] M.‐S. Xiao , Y. Ai , J. E. Wilusz , Trends Cell Biol. 2020, 30, 226. 31973951 10.1016/j.tcb.2019.12.004 PMC7069689

[7] L. S. Kristensen , M. S. Andersen , L. V. W. Stagsted , K. K. Ebbesen , T. B. Hansen , J. Kjems , Nat. Rev. Genet. 2019, 20, 675. 31395983 10.1038/s41576-019-0158-7

[8] X. Li , L.i Yang , L.‐L. Chen , Mol. Cell 2018, 71, 428. 30057200 10.1016/j.molcel.2018.06.034

[9] Y. Enuka , M. Lauriola , M. E. Feldman , A. Sas‐Chen , I. Ulitsky , Y. Yarden , Nucleic Acids Res. 2016, 44, 1370. 26657629 10.1093/nar/gkv1367 PMC4756822

[10] I. Legnini , G. Di Timoteo , F. Rossi , M. Morlando , F. Briganti , O. Sthandier , A. Fatica , T. Santini , A. Andronache , M. Wade , P. Laneve , N. Rajewsky , I. Bozzoni , Mol. Cell 2017, 66, 22. 28344082 10.1016/j.molcel.2017.02.017 PMC5387670

[11] N. R. Pamudurti , O. Bartok , M. Jens , R. Ashwal‐Fluss , C. Stottmeister , L. Ruhe , M. Hanan , E. Wyler , D. Perez‐Hernandez , E. Ramberger , S. Shenzis , M. Samson , G. Dittmar , M. Landthaler , M. Chekulaeva , N. Rajewsky , S. Kadener , Mol. Cell 2017, 66, 9. 28344080 10.1016/j.molcel.2017.02.021 PMC5387669

[12] E. C. S. Lee , S. A. M. Elhassan , G. P. L. Lim , W. H. Kok , S. W. Tan , E.e N. Leong , S. H. Tan , E. W. L. Chan , S. K. Bhattamisra , R. Rajendran , M. Candasamy , Biomed. Pharmacother. 2019, 111, 198. 30583227 10.1016/j.biopha.2018.12.052

[13] S.‐Y. Wen , J. Qadir , B. B. Yang , Trends Mol. Med. 2022, 28, 405. 35379558 10.1016/j.molmed.2022.03.003

[14] M. S. Ebert , P. A. Sharp , RNA 2010, 16, 2043. 20855538 10.1261/rna.2414110 PMC2957044

[15] N. Wu , Z. Yuan , K. Y. Du , L. Fang , J. Lyu , C. Zhang , A. He , E. Eshaghi , K. Zeng , J. Ma , W. W. Du , B. B. Yang , Cell Death Differ. 2019, 26, 2758. 31092884 10.1038/s41418-019-0337-2 PMC7224378

[16] N. Wu , J. Xu , W. W. Du , X. Li , F. M. Awan , F. Li , S. Misir , E. Eshaghi , J. Lyu , L.e Zhou , K. Zeng , A. Adil , S. Wang , B. B. Yang , Mol. Ther. 2020, 29, 1138. 33279723 10.1016/j.ymthe.2020.12.004 PMC7934790

[17] W. W. Du , W. Yang , E. Liu , Z. Yang , P. Dhaliwal , B. B. Yang , Nucleic Acids Res. 2016, 44, 2846. 26861625 10.1093/nar/gkw027 PMC4824104

[18] W. W. Du , X. Li , J. Ma , L. Fang , N. Wu , F. Li , P. Dhaliwal , W. Yang , A. J. Yee , B. B. Yang , Molecular Ther. –Nucleic Acids 2022, 27, 276. 10.1016/j.omtn.2021.11.027 PMC8718830 35024241

[19] W. W. Du , J. Xu , W. Yang , N. Wu , F. Li , L.e Zhou , S. Wang , X. Li , A. T. He , K. Y. Du , K. Zeng , J. Ma , J. Lyu , C. Zhang , C. Zhou , K. Maksimovic , B. B. Yang , Circ. Res. 2021, 129, 568. 34261347 10.1161/CIRCRESAHA.120.318364

[20] W. W. Du , W. Yang , X. Li , F. M. Awan , Z. Yang , L. Fang , J. Lyu , F. Li , C. Peng , S. N. Krylov , Y. Xie , Y. Zhang , C. He , N. Wu , C. Zhang , M. Sdiri , J. Dong , J. Ma , C. Gao , S. Hibberd , B. B. Yang , Oncogene 2018, 37, 5829. 29973691 10.1038/s41388-018-0369-y

[21] W. W. Du , W. Yang , X. Li , L. Fang , N. Wu , F. Li , Y.u Chen , Q. He , E. Liu , Z. Yang , F. M. Awan , M. Liu , B. B. Yang , Mol. Ther. 2020, 28, 1287. 32229309 10.1016/j.ymthe.2020.03.002 PMC7210749

[22] L. Fang , W. W. Du , J. Lyu , J. Dong , C. Zhang , W. Yang , A. He , Y. S. S. Kwok , J. Ma , N. Wu , F. Li , F. M. Awan , C. He , B. L. Yang , C. Peng , H. J. Mackay , A. J. Yee , B. B. Yang , Cell Death Differ. 2018, 25, 2195. 29795334 10.1038/s41418-018-0115-6 PMC6261950

[23] A. T. He , J. Liu , F. Li , B. B. Yang , Signal Transduction Targeted Ther. 2021, 6, 185. 10.1038/s41392-021-00569-5 PMC8137869 34016945

[24] Q. Yang , F. Li , A. T. He , B. B. Yang , Molecular therapy : the journal of the American Society of Gene Therapy 2021, 29, 1683. 33484969 10.1016/j.ymthe.2021.01.018 PMC8116570

[25] E. Ford , M. Ares , Proc. Natl. Acad. Sci. USA 1994, 91, 3117. 7512723 10.1073/pnas.91.8.3117 PMC43526

[26] H. Ho‐Xuan , P. Glazar , C. Latini , K. Heizler , J. Haase , R. Hett , M. Anders , F. Weichmann , A. Bruckmann , D. Van den Berg , S. Hüttelmaier , N. Rajewsky , C. Hackl , G. Meister , Nucleic Acids Res. 2020, 48, 10368. 32955563 10.1093/nar/gkaa704 PMC7544230

[27] R. A. Wesselhoeft , P. S. Kowalski , D. G. Anderson , Nat. Commun. 2018, 9, 2629. 29980667 10.1038/s41467-018-05096-6 PMC6035260

[28] S. Aufiero , Y. J. Reckman , Y. M. Pinto , E. E. Creemers , Nat. Rev. Cardiol. 2019, 16, 503. 30952956 10.1038/s41569-019-0185-2

[29] D. Liang , J. E. Wilusz , Genes Dev. 2014, 28, 2233. 25281217 10.1101/gad.251926.114 PMC4201285

[30] F. X. R. Sutandy , S. Ebersberger , L.u Huang , A. Busch , M. Bach , H.‐S. Kang , J. Fallmann , D. Maticzka , R. Backofen , P. F. Stadler , K. Zarnack , M. Sattler , S. Legewie , J. König , Genome Res. 2018, 28, 699. 29643205 10.1101/gr.229757.117 PMC5932610

[31] S. Das , A. R. Krainer , Mol. Cancer Res. 2014, 12, 1195. 24807918 10.1158/1541-7786.MCR-14-0131 PMC4163531

[32] O. Anczuków , M. Akerman , A. Cléry , J. Wu , C. Shen , N. H. Shirole , A. Raimer , S. Sun , M. A. Jensen , Y. Hua , F. H.‐T. Allain , A. R. Krainer , Mol. Cell 2015, 60, 105. 26431027 10.1016/j.molcel.2015.09.005 PMC4597910

[33] Y. Che , L. Fu , J. Cancer 2020, 11, 3502. 32284746 10.7150/jca.42645 PMC7150454

[34] B. Yang , original wb‐ms ‐Aug 10, 2022.pdf. figshare. Figure. 10.6084/m9.figshare.20482158.v1 2022

