# Supplementary material for: An Improved Model for Circular RNA Overexpression: Using the Actin Intron Reveals High Circularization Efficiency
Source: Adv Genet (Hoboken). 2022 Oct 9;4(3):2200019. doi: 10.1002/ggn2.202200019 (PMC10520416; doi:10.1002/ggn2.202200019)
Supplement: Supplementary file 1 — Supporting Information [file GGN2-4-2200019-s001.pdf]

ADVANCED GENETICS
Open Access

Supporting Information

for *Advanced Genetics*, DOI 10.1002/ggn2.202200019

An Improved Model for Circular RNA Overexpression: Using the Actin Intron Reveals High Circularization Efficiency

*Feiya Li, Juanjuan Lyu, Yang Yang, Qiwei Yang, Cristian Santos and Burton B. Yang**

Table S1. Beta-actin Intron sequence used to generate circRNA expression constructs.

Gtgagtggcccgctacctcttctggtggccgcctccctccttcctggcct

cccggagctgcgccctttctcactggttctctcttctgccgttttccgtag

gactctcttctctgacctgagtctcctttggaactctgcaggttctatttgc

tttttcccagatgagctctttttctggtgtttgtctctctgactaggtgtctg

agacagtgttgtgggtgtaggtactaacactggctcgtgtgacaaggc

catgaggctggtgtaaaagtgtgtattaagtaggcgcacagtaggtct

gaacagactccccatcccaagaccccagcgcggccttggacacttagc

cgtgttctttgcactttctgcatgtccccgtctggcctggctgtccccag

tggcttccccagtgtgacatggtgcatctctgccttacag

Beta-actin intron sets were used to undergo back-splicing resulting in transcript circularization to form circular RNAs. Intron 15, 15 nucleotides from both splicing sites labelled in purple (underlined purple); Intron 30, 30 nucleotides from both splicing sites labelled by red underline; Intron 60, 60 nucleotides from both splicing sites labelled by blue underline; Intron 100, 100 nucleotides from both splicing sites labelled by green underline; Intron 180, 180 nucleotides from both splicing sites labelled by yellow underline.

Table S2. siRNA sequences

1.hu-si-SRSF1
5′   gcaucuacgugggguaacuuuu
3′ ggcguagaugcacccauugaa

2.hu-si-SRSF3
5′   gguguacaggaaauuacuuuu
3′ ggccacauguccuuuaaugaa

3.hu-si-SRSF4
5′   gcauaaaaguaagagcaaauu
3′ ggcguauuuucauucucguuu

4.hu-si-U2AF2
5′   ggcagcucaacgagaauaauu
3′ ggccgucgaguugcucuuauu

5.hu-mu-si-SRSF1
5′   gccgcaucuacgugggguaauu
3′ ggcggcguagaugcacccauu

6.hu-mu-si-SRSF3
5′   cgaucuaggucaaaugaaauu
3′ ccgcuagauccaguuuacuuu

7.hu-mu-si-SRSF4
5′   gacgcaguggauaugguuauu
3′ ggcugcgucaccuauaccaau

8.hu-mu-si-U2AF2
5′   ggcagcucaacgagaauaauu
3′ ggccgucgaguugcucuuauu

Table S3. Mass spectrophotometry results providing potential explanation for intron 180 low circularization efficiency. A specific binding of SRSF4 splicing to intron 180 might provide potential explanation of low circularization efficiency in a longer intron.

Mass spectrophotometry results showing splicing factors binding to introns

Accession Number	Alternate ID	Molecular Weight	CP11	CP17	CP18	CP19
P23246 (+1)	SFPQ	76 kDa	7	10	10	4
P26368 (+1)	U2AF2	54 kDa	3	2	10	2
Q07955 (+2)	SRSF1	28 kDa	3	3	10	3
Q6P2Q9	PRPF8	274 kDa	5	6	5	2
O43143	DHX15	91 kDa	4	5	3	3
P84103 (+1)	SRSF3	19 kDa	2	4	7	2
Q9UHX1-2	PUF60	58 kDa			2	2
Q13435	SF3B2	100 kDa	3	1	3	2
Q13243	SRSF5	31 kDa	2	2	4	3
O75494 (+5)	SRSF10	31 kDa	3	5	3	
Q15393	SF3B3	136 kDa	4	2	1	1
O75533	SF3B1	146 kDa	2	1	3	2
Q16629 (+3)	SRSF7	27 kDa	3	4	6	2
Q15427	SF3B4	44 kDa	2	2	1	1
Q01130 (+1)	SRSF2	25 kDa	1		3	
Q08170 (+2)	SRSF4	57 kDa				2
Q12874	SF3A3	59 kDa	1			1
P0DN76 (+3)	U2AF1L5	28 kDa			1	
Q13242	SRSF9	26 kDa			1	

Green arrow show splicing factor potentially interacting with Actin intron 180

Table S4. Plasmid sequences

CP11-circPtprf-actin15-2CMV
tagttattaatagtaatcaat—CMV (1-619 bp)---cc**gctagc**gctaccggactc**agatctcgag**(XhoI)
atctctgccttacagtgcgccgcgtggctcctcgtttctccatccctcccagcagccaggaggtgatgccaggcggcagcgtgaacctgacatgcgtggcagtgggtgcacccatgccctacgtgaagtggatgatgggggccgaggagctc
accaaggaggatgagatgccagttggccgcaacgtcctggagctcagcaatgtcgtacgctctgccaactacacctgtgtggccatctcctcgctgggcatgatcgaggccacagcccaggtcacagtgaaaggtgagtggcccgcta
ggatcc(BamHI)tagttattaatagtaatcaat—CMV (1-617 bp)--cc**gctagc**gctaccggactc**agatctcg**
accggt(AgeI)cgccacc**ATG**gtgagca (667-4740bp of pEGFP-N1)
CP12-circPtprf-actin30-2CMV
tctcgagagtgtgacatggtgcatctctgccttacagtgcgccgcgtggctcctcgtttctccatccctcccagcagccaggaggtgatgccaggcggcagcgtgaacctgacatgcgtggcagtgggtgcacccatgccctacgtgaagtgg
atgatgggggccgaggagctcaccaaggaggatgagatgccagttggccgcaacgtcctggagctcagcaatgtcgtacgctctgccaactacacctgtgtggccatctcctcgctgggcatgatcgaggccacagcccaggtcacagtga
aaggtgagtggcccgctacctcttctggtggcc**ggatcc**t
CP13-circPtprf-actin60-2CMV
tctcgagtctggcctggctgtccccagtggcttccccagtgtgacatggtgcatctctgccttacagtgcgccgcgtggctcctcgtttctccatccctcccagcagccaggaggtgatgccaggcggcagcgtgaacctgacatgcgtggcag
tgggtgcacccatgccctacgtgaagtggatgatgggggccgaggagctcaccaaggaggatgagatgccagttggccgcaacgtcctggagctcagcaatgtcgtacgctctgccaactacacctgtgtggccatctcctcgctgggcat
gatcgaggccacagcccaggtcacagtgaaaggtgagtggcccgctacctcttctggtggccgcctccctccttcctggcctcccggagctg**ggatcc**t
CP14circPtprf-actin100-2CMV
tctcgagacacttagccgtgttctttgcactttctgcatgtcccccgtctggcctggctgtccccagtggcttccccagtgtgacatggtgcatctctgccttacagtgcgccgcgtggctcctcgtttctccatccctcccagcagccaggaggtg
atgccaggcggcagcgtgaacctgacatgcgtggcagtgggtgcacccatgccctacgtgaagtggatgatgggggccgaggagctcaccaaggaggatgagatgccagttggccgcaacgtcctggagctcagcaatgtcgtacgctct
gccaactacacctgtgtggccatctcctcgctgggcatgatcgaggccacagcccaggtcacagtgaaaggtgagtggcccgctacctcttctggtggccgcctccctccttcctggcctcccggagctgcgccctttctcactggttctctctt
ctgccgttttccgta**ggatcc**t
CP15-circPtprf-actin180-2CMV
tctcgagatgaggctggtgtaaagcggccttggagtgtgtattaagtaggcgcacagtaggtctgaacagactccccatcccaagaccccagcacacttagccgtgttctttgcactttctgcatgtcccccgtctggcctggctgtccccagt
ggcttccccagtgtgacatggtgcatctctgccttacagtgcgccgcgtggctcctcgtttctccatccctcccagcagccaggaggtgatgccaggcggcagcgtgaacctgacatgcgtggcagtgggtgcacccatgccctacgtgaagt
ggatgatgggggccgaggagctcaccaaggaggatgagatgccagttggccgcaacgtcctggagctcagcaatgtcgtacgctctgccaactacacctgtgtggccatctcctcgctgggcatgatcgaggccacagcccaggtcacagt
gaaaggtgagtggcccgctacctcttctggtggccgcctccctccttcctggcctcccggagctgcgccctttctcactggttctctcttctgccgttttccgtaggactctcttctctgacctgagtctcctttggaactctgcaggttctatttgct
ttttcccagatgagctctttttctggtgtttg**ggatcc**t
CP16-circPtprf-actinT4-2CMV
ctcgagaaatctgataaatggaattggttctacataaatgcctaacgactatccctttggggagtagggtcaagtgactcgaaacgatagacaacttgctttaacaagttggagatatagtctgctctgcatggtgacatgcagctggatata
attccggggtaagattaacgaccttatctgaacataatgtgcgccgcgtggctcctcgtttctccatccctcccagcagccaggaggtgatgccaggcggcagcgtgaacctgacatgcgtggcagtgggtgcacccatgccctacgtgaag
tggatgatgggggccgaggagctcaccaaggaggatgagatgccagttggccgcaacgtcctggagctcagcaatgtcgtacgctctgccaactacacctgtgtggccatctcctcgctgggcatgatcgaggccacagcccaggtcacag
tgaaagtaattgaggcctgagtataaggtgacttatacttgtaatctatctaaacggggaacctctctagtagacaatcccgtgctaaattgtaggacttgccctttaataaatacttctatatttaaagaggtatttatgaaaagcggaattt
atcagattaaaaatactttaaacaataaagtatatgta**ggatcc**a
CP17-circPTPRF-actin15-mut-2CMV (NC32.circPTPRF-actin15-mut)
tctcgagatctctgccttacagtgcgccgcgtggctcctcgtttctccatccctcccagcagccaggaggtgatgccaggcggcagcgtgaacctgacatgcgtggcagtgggtgcacccatgccctacgtgaagtggatgatgggggccga
ggagctcaccaacctccta**ACGCGT**gtccaaccaacgttgcacctggagctcagcaatgtcgtacgctctgccaactacacctgtgtggccatctcctcgctgggcatgatcgaggccacagcccaggtcacagtgaaaggtgagtggc
ccgcta**ggatcct**
CP18.circPtprf-actin100-mut-2CMV
tctcgagacacttagccgtgttctttgcactttctgcatgtcccccgtctggcctggctgtccccagtggcttccccagtgtgacatggtgcatctctgccttacagtgcgccgcgtggctcctcgtttctccatccctcccagcagccaggaggtg
atgccaggcggcagcgtgaacctgacatgcgtggcagtgggtgcacccatgccctacgtgaagtggatgatgggggccgaggagctcaccaacctccta**ACGCGT**gtccaaccaacgttgcacctggagctcagcaatgtcgtacgctc
tgccaactacacctgtgtggccatctcctcgctgggcatgatcgaggccacagcccaggtcacagtgaaaggtgagtggcccgctacctcttctggtggccgcctccctccttcctggcctcccggagctgcgccctttctcactggttctctct
tctgccgttttccgta**ggatcc**t
CP19-circPTPRF-actin180-mut-2CMV (NC34.circPTPRF-actin180-mut)
tctcgagatgaggctggtgtaaagcggccttggagtgtgtattaagtaggcgcacagtaggtctgaacagactccccatcccaagaccccagcacacttagccgtgttctttgcactttctgcatgtcccccgtctggcctggctgtccccagt
ggcttccccagtgtgacatggtgcatctctgccttacagtgcgccgcgtggctcctcgtttctccatccctcccagcagccaggaggtgatgccaggcggcagcgtgaacctgacatgcgtggcagtgggtgcacccatgccctacgtgaagt
ggatgatgggggccgaggagctcaccaacctccta**ACGCGT**gtccaaccaacgttgcacctggagctcagcaatgtcgtacgctctgccaactacacctgtgtggccatctcctcgctgggcatgatcgaggccacagcccaggtcaca
gtgaaaggtgagtggcccgctacctcttctggtggccgcctccctccttcctggcctcccggagctgcgccctttctcactggttctctcttctgccgttttccgtaggactctcttctctgacctgagtctcctttggaactctgcaggttctatttg
ctttttcccagatgagctctttttctggtgtttg**ggatcc**t
CP20-circBrf2-actin15-2CMV
tctcgagatctctgccttacaggtctccggcgagtgagagacctttgtcgagttctgcagttgccaccaacatttgaggataccgcggttgcctactaccaacaggcatatcggcactctggcatccgagcggccaggctgcaaaagaagga
ggtgttggttgggtgctgcgtcttaatcacctgccgacagcataactggcccctaacaatgggggccatctgcacgctgttgtatgcagatttggatgtgttttctagcacttacatgcagatagtgaagctcctgggactggatgtgccatctc
tgtgcttggcagaactggtgaagacctattgcagcaggtgagtggcccgcta**ggatcct**
CP21-circBrf2-actin30-2CMV
tctcgagagtgtgacatggtgcatctctgccttacaggtctccggcgagtgagagacctttgtcgagttctgcagttgccaccaacatttgaggataccgcggttgcctactaccaacaggcatatcggcactctggcatccgagcggccagg
ctgcaaaagaaggaggtgttggttgggtgctgcgtcttaatcacctgccgacagcataactggcccctaacaatgggggccatctgcacgctgttgtatgcagatttggatgtgttttctagcacttacatgcagatagtgaagctcctgggac
tggatgtgccatctctgtgcttggcagaactggtgaagacctattgcagcaggtgagtggcccgctacctcttctggtggcc**ggatcc**t
CP22-circBrf2-actin60-2CMV
tctcgagtctggcctggctgtccccagtggcttccccagtgtgacatggtgcatctctgccttacaggtctccggcgagtgagagacctttgtcgagttctgcagttgccaccaacatttgaggataccgcggttgcctactaccaacaggcat
atcggcactctggcatccgagcggccaggctgcaaaagaaggaggtgttggttgggtgctgcgtcttaatcacctgccgacagcataactggcccctaacaatgggggccatctgcacgctgttgtatgcagatttggatgtgttttctagca
cttacatgcagatagtgaagctcctgggactggatgtgccatctctgtgcttggcagaactggtgaagacctattgcagcaggtgagtggcccgctacctcttctggtggccgcctccctccttcctggcctcccggagctg**ggatcc**t
CP23-circBrf2-actin100-2CMV
tctcgagacacttagccgtgttctttgcactttctgcatgtcccccgtctggcctggctgtccccagtggcttccccagtgtgacatggtgcatctctgccttacaggtctccggcgagtgagagacctttgtcgagttctgcagttgccaccaac
atttgaggataccgcggttgcctactaccaacaggcatatcggcactctggcatccgagcggccaggctgcaaaagaaggaggtgttggttgggtgctgcgtcttaatcacctgccgacagcataactggcccctaacaatgggggccatct
gcacgctgttgtatgcagatttggatgtgttttctagcacttacatgcagatagtgaagctcctgggactggatgtgccatctctgtgcttggcagaactggtgaagacctattgcagcaggtgagtggcccgctacctcttctggtggccgcct
ccctccttcctggcctcccggagctgcgccctttctcactggttctctcttctgccgttttccgta**ggatcc**t
CP39-circBrf2-actin180-2CMV
atgaggctggtgtaaagcggccttggagtgtgtattaagtaggcgcacagtaggtctgaacagactccccatcccaagaccccagcacacttagccgtgttctttgcactttctgcatgtcccccgtctggcctggctgtccccagtggcttcc
ccagtgtgacatggtgcatctctgccttacaggtctccggcgagtgagagacctttgtcgagttctgcagttgccaccaacatttgaggataccgcggttgcctactaccaacaggcatatcggcactctggcatccgagcggccaggctgca
aaagaaggaggtgttggttgggtgctgcgtcttaatcacctgccgacagcataactggcccctaacaatgggggccatctgcacgctgttgtatgcagatttggatgtgttttctagcacttacatgcagatagtgaagctcctgggactggat
gtgccatctctgtgcttggcagaactggtgaagacctattgcagcaggtgagtggcccgctacctcttctggtggccgcctccctccttcctggcctcccggagctgcgccctttctcactggttctctcttctgccgttttccgtaggactctcttc
tctgacctgagtctcctttggaactctgcaggttctatttgctttttcccagatgagctctttttctggtgtttg
CP40-circBrf2-T4-2CMV
aaatctgataaatggaattggttctacataaatgcctaacgactatccctttggggagtagggtcaagtgactcgaaacgatagacaacttgctttaacaagttggagatatagtctgctctgcatggtgacatgcagctggatataattccg
gggtaagattaacgaccttatctgaacataatggtctccggcgagtgagagacctttgtcgagttctgcagttgccaccaacatttgaggataccgcggttgcctactaccaacaggcatatcggcactctggcatccgagcggccaggctg
caaaagaaggaggtgttggttgggtgctgcgtcttaatcacctgccgacagcataactggcccctaacaatgggggccatctgcacgctgttgtatgcagatttggatgtgttttctagcacttacatgcagatagtgaagctcctgggactg
gatgtgccatctctgtgcttggcagaactggtgaagacctattgcagcagtaattgaggcctgagtataaggtgacttatacttgtaatctatctaaacggggaacctctctagtagacaatcccgtgctaaattgtaggacttgccctttaat
aaatacttctatatttaaagaggtatttatgaaaagcggaatttatcagattaaaaatactttaaacaataaagtatatgta

Table S5. Primer sequences

31.71.hu-circ-PTPRF-R	5′ catcacctcctggctgctgggagg
31.72.hu-circ-PTPRF-F	5′ cctgtgtggccatctcctcgctgg
31.73.hu-circ-BRF2-R	5′ gttggtggcaactgcagaactcga
31.74.hu-circ-BRF2-F	5′ ggatgtgccatctctgtgcttggc
32.51.Hu.cir.Ptprf-actin-intron-F.	5′ tgcatctctgccttacag tgcgccgcgtggctcctc
32.52.Hu.cir.Ptprf-actin-intron-R	5′ aggtagcgggccactcac ctttcactgtgacctggg
32.53.Hu.cir.Brf2-actin-intron-F.	5′ tgcatctctgccttacag gtctccggcgagtgagag
32.54.Hu.cir.Brf2-actin-intron-R	5′ aggtagcgggccactcac ctgctgcaataggtcttc
33.24.Hu.cir.Ptprf-T4-intron-F.	5′ ccttatctgaacataatg tgcgccgcgtggctcctc
33.25.Hu.cir.Ptprf-T4-intron-R	5′ atactcaggcctcaatta ctttcactgtgacctggg
33.26.Hu.cir.Brf2-T4-intron-F.	5′ ccttatctgaacataatg gtctccggcgagtgagag
33.27.Hu.cir.Brf2-T4-intron-R	5′ atactcaggcctcaatta ctgctgcaataggtcttc
19-19-human-U6RNAf	5′ gtgct cgcttcggca gcacatatac
19-20.humanU6RNAr	5′ aa aaa tat gg aa cgc ttc acga atttg

32.17.T4-intron-F-XhoI

5′gggcccgc**ctcgag**aaatctgataaatggaattggttctacataaatgcctaacgactatccctttggggagtagggtcaagtgactcgaaacgatagacaacttgctttaacaagttggagatatagtctgctctgcatggt gacatgcagctggatataattccggggtaagattaacgaccttatctgaacataatg

32.18.T4-intron-F-BamHI

5′gcatgcat**ggatcc**tacatatactttattgtttaaagtatttttaatctgataaattccgcttttcataaatacctctttaaatatagaagtatttattaaagggcaagtcctacaatttagcacgggattgtctactagagaggtt ccccgtttagatagattacaagtataagtcaccttatactcaggcctcaatta

32.19.Actin-intron-F-XhoI

5′gggcccgc ctcgag

atgaggctggtgtaaagcggccttggagtgtgtattaagtaggcgcacagtaggtctgaacagactccccatcccaagaccccagcacacttagccgtgttctttgcactttctgcatgtcccccgtctggcctggctgtccccag tggcttccccagtgtgacatggtgcatctctgccttacag

32.20.Actin-intron-R-BamHI

5′gcatgcat**ggatcc**caaacaccagaaaaagagctcatctgggaaaaagcaaatagaacctgcagagttccaaaggagactcaggtcagagaagagagtcctacggaaaacggcagaagagagaaccagtgagaaa gggcgcagctccgggaggccaggaaggagggaggcggccaccagaagaggtagcgggccactcac

32.45.T4-intron-F2-XhoI	5′gggcccgc**ctcgag**aaat
32.46.T4-intron-R2-BamHI	5′gcatgcat**ggatcc**taca
32.47.Actin-intron-F2-XhoI	5′gggcccgc ctcgag atga
32.48.Actin-intron-R2-BamHI	5′gcatgcat**ggatcc**caaa
32.49.Actin-intron100-F-XhoI	5′gggcccgc ctcgag acacttagccgtgttctt
32.50.Actin-intron100-R-BamHI	5′gcatgcat**ggatcc** tacggaaaacggcagaag
32.67.Actin-intron15-F-XhoI	5′ cggggcccgc ctcgag **atctctgccttacag**
32.68.Actin-intron15-R-BamHI	5′ cggcatgcat **ggatcc** gtgagtggcccgcta
32.69.Actin-intron30-F-XhoI	5′ cggggcccgc ctcgag agtgtgacatggtgc**atc**
32.70.Actin-intron30-R-BamHI	5′ cggcatgcat **ggatcc** ctacctcttctggtggcc
32.71.Actin-intron60-F-XhoI	5′ cggggcccgc ctcgag tctggcctggctgtcccc
32.72.Actin-intron60-R-BamHI	5′ cggcatgcat **ggatcc** cctggcctcccggagctg
33.5.Actin-intron100-R-BglII	5′ gcatgcat **agatct** tacggaaaacggcagaag
33.6.humu.actin-gene-FXhoI	5′ cggggcccgc ctcgag atggatgatgatatcgccgcgctcgt
33.7.humu.actin-gene-RBamHI	5′ cggcatgcat **ggatcc** cgctcggtgaggatcttcatgagg (~1.1 kb)
32.68.Actin-intron15-R-BamHI	5′ cggcatgcat **ggatcc** tagcgggccactcac
32.70.Actin-intron30-R-BamHI	5′ cggcatgcat **ggatcc** ggccaccagaagaggtag
32.72.Actin-intron60-R-BamHI	5′ cggcatgcat **ggatcc** cagctccgggaggccagg
33.20.humu.actin216-F	5′ Ggtgggcatgggtcagaaggattc
33.21.humu.actin414-R	5′ caggggggcctcggtcagcagcac
33.36.Hu.actin-intron-F	5′ cacagcgcgcccggctattctcgc (to combine with 33.7.humu.actin-gene-RBamHI, 1.2 kb)
33.37.Mu.actin-intron-F	5′ ggccccgaggtgactatagccttc (to combine with 33.7.humu.actin-gene-rbamhi, 1.2 kb)

33.42.Actin-intron-round2F

5′ acacttagccgtgttctttgcactttctgcatgtcccccgtctggcctggctgtccccagtggcttccccagtgtgacatggtgcatctctgccttacag

33.43.Actin-intron-round2R

5′ tacggaaaacggcagaagagagaaccagtgagaaagggcgcagctccgggaggccaggaaggagggaggcggccaccagaagaggtagcgggccactcac

34.9.Intron-probe-F	5′ tcaccaacctccta**ACGC**
34.10.Intron-probe-R	5′ ctccaggtgcaacgttgg (large scale)
37.1.Hu-cirSlc8A1-F	5′ tgccagaggtggaggggaggat
37.2.Hu-cirSlc8A1-R	5′ atcgccgcatgttgtacatgac
39.27.hu-mu-Srsf1-F	5′ tgccgcatctacgtgggtaac
39.28.hu-mu-Srsf1-R	5′ gaagggcggtcccccgcggcg (123 bp)
39.29.hu-mu-Srsf3-F	5′ ctttctagagataggagaagaga
39.30.hu-mu-Srsf3-F	5′ ttcctttcatttgacctagatcg (107 bp)
39.31.hu-mu-SRSF4-F	5′ ggacgcagtggatatggttat
39.32.hu-mu-SRSF4-R	5′ cgacttgacaaattctccacaa (98 bp)
39.33.hu-mu-U2AF2-F	5′ aaacaagagcgggacaaggag
39.34.hu-mu-U2AF2-R	5′ cgctggtcccggttgcgccg (113 bp)

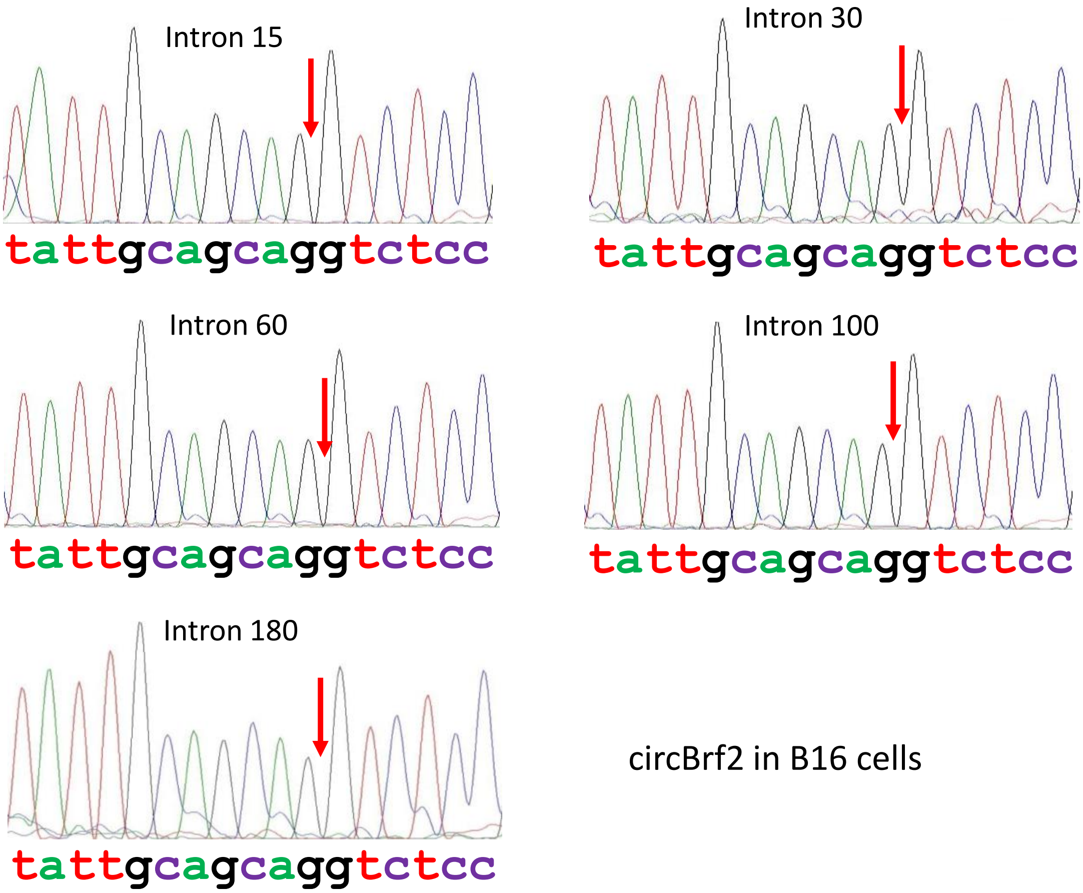

Supplementary Figure S1. Sanger sequencing of the “head-to-tail” junctions.
B16 cells were transfected with circBrf2 plasmids with different introns. Collected samples were PCR and resolved on gel and gels were cut and purified sent out for junction detection. Red arrows: “head-to-tail” junction points.

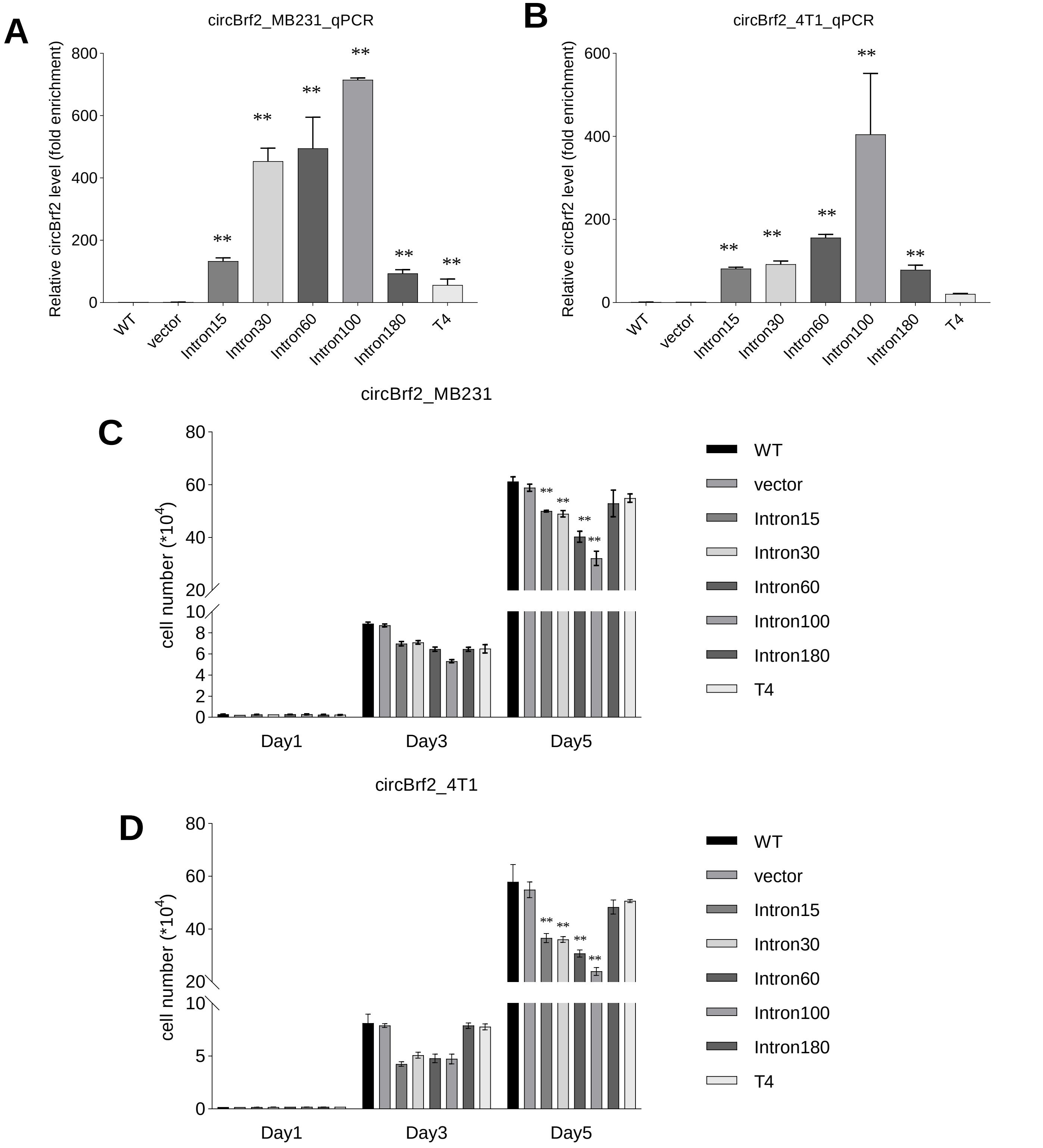

Supplementary Figure S2. Functional assays of the constructs.
Human breast cancer cell line MDA-MB-231 **(A)** and mouse breast cancer cell line 4T1 **(B)** were transfected with different constructs of circBrf2 generated using different intron sets as shown. Significantly higher levels of circBrf2 circularization was detected. In cell proliferation assay on day 5, decrease in cell proliferation was detected with the circBrf2 constructs expressed in MDA-MB-231 **(C)** and 4T1 cells **(D)** . **p<0.01, One-way ANOVA, compared to vector.

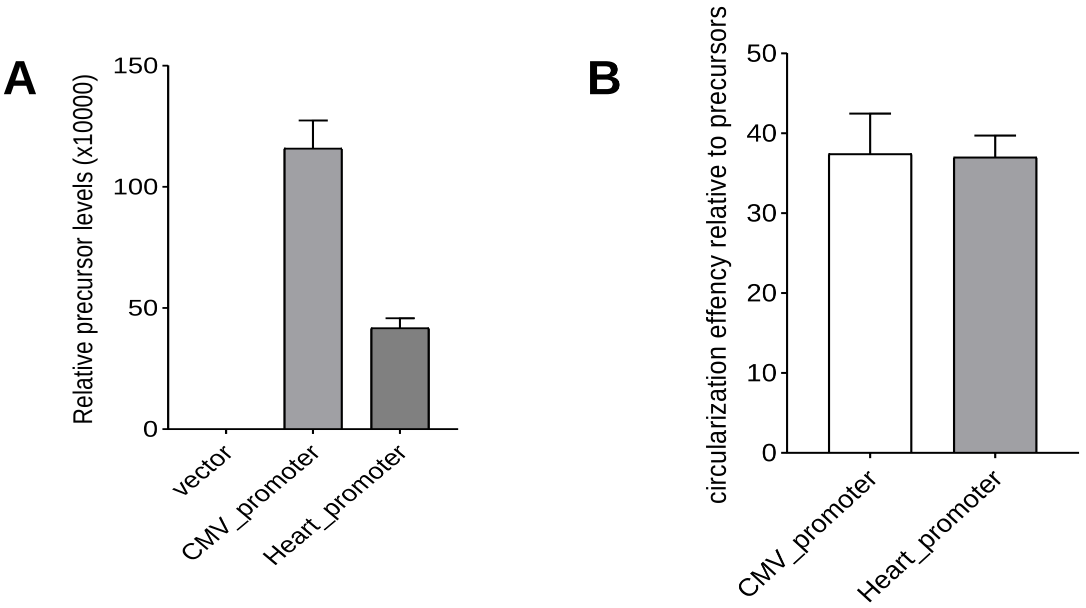

CP52.hucircSlc8A1

gcgttacataacttacggtaaatggcccgcctggctgaccgcccaacgacccccgcccattgacgtcaataatgacgtatgttcccatagtaacgccaatagggactttccattgacgtcaatgggtggagtatttacggta
aactgcccacttggcagtacatcaagtgtatcatatgccaagtacgccccctattgacgtcaatgacggtaaatggcccgcctggcattatgcccagtacatgaccttatgggactttcctacttggcagtacatctacgtatt
agtcatcgctattaccatggtgatgcggttttggcagtacatcaatgggcgtggatagcggtttgactcacggggatttccaagtctccaccccattgacgtcaatgggagtttgttttggcaccaaaatcaacgggactttcc
aaaatgtcgtaacaactccgccccattgacgcaaatgggcggtaggcgtgtacggtgggaggtctatataagcagagctg

gtcgacacacttagccgtgttctttgcactttctgcatgtcccccgtctggcctggctgtccccagtggcttccccagtgtgacatggtgcatctctgccttacag

gttgtgacagttggaagtgtc**atg**tacaacatgcggcgattaagtctttcacccaccttttcaatgggatttcatctgttagttactgtgagtctcttattttcccatgtggaccatgtaattgctgagacagaaatggaaggagaaggaaatgaaactggtg
aatgtactggatcatattactgtaagaaaggggtgattttgcccatttgggaaccccaagacccttcttttggggacaaaattgctagagctactgtgtattttgtggccatggtctacatgtttcttggagtctctatcatagctgatcggttcatgtcctctat
agaagtcatcacatctcaagaaaaagaaataaccataaagaaacccaatggagagaccaccaagacaactgtgaggatctggaatgaaacagtttctaacctgaccttgatggccctgggatcttctgctcctgagattctcctttcagtaattgaagtg
tgtggccataacttcactgcaggagacctcggtcctagcaccatcgtgggaagtgctgcattcaatatgttcatcattattgcactctgtgtttatgtggtgcctgacggagagacaaggaagattaagcatttgcgtgtcttctttgtgacagcagcctggag
catctttgcctacacctggctttacattattttgtctgtcatatctcctggtgttgtggaggtctgggaaggtttgcttactttcttcttctttcccatctgtgttgtgttcgcttgggtagcggataggagacttctgttttacaagtatgtctacaagaggtatcga
gctggcaagcagagggggatgattattgaacatgaaggagacaggccatcttctaagactgaaattgaaatggacgggaaagtggtcaattctcatgttgaaaatttcttagatggtgctctggttctggaggtggatgagagggaccaagatgatgaag
aagctaggcgagaaatggctaggattctgaaggaacttaagcagaagcatccagataaagaaatagagcaattaatagaattagctaactaccaagtcctaagtcagcagcaaaaaagtagagcattttatcgcattcaagctactcgcctcatgactg
gagctggcaacattttaaagaggcatgcagctgaccaagcaaggaaggctgtcagcatgcacgaggtcaacactgaagtgactgaaaatgaccctgttagtaagatcttctttgaacaagggacatatcagtgtctggagaactgtggtactgtggccct
taccattatccgcagaggtggtgatttgactaacactgtgtttgttgacttcagaacagaggatggcacagcaaatgctgggtctgattatgaatttactgaaggaactgtggtgtttaagcctggtgatacccagaaggaaatcagagtgggtatcataga
tgatgatatctttgaggaggatgaaaatttccttgtgcatctcagcaatgtcaaagtatcttctgaagcttcagaagatggcatactggaagccaatcatgtttctacacttgcttgcctcggatctccctccactgccactgtaactatttttgatgatgacca
cgcaggcatttttacttttgaggaacctgtgactcatgtgagtgagagcattggcatcatggaggtgaaagtattgagaacatctggagctcgaggaaatgttatcgttccatataaaaccatcgaagggactgccagaggtggaggggaggattttgagg
acacttgtggagagctcgaattccagaatgatgaaattgt

gtgagtggcccgctacctcttctggtggccgcctccctccttcctggcctcccggagctgcgccctttctcactggttctctcttctgccgttttccgta**ggatcc**

CP72.hucircSlc8A1-heart

ccagttgttcaactcacccttcagattaaaaataactgaggtaagggcctgggtaggggaggtggtgtgagacgctcctgtctctcctctatctgcccatcggccctttggggaggaggaatgtgcccaaggactaaaaaaaggccatggagccagaggg
gcgagggcaacagacctttcatgggcaaaccttggggccctgctgtcctcctgtcacctccagagccaagggatcaaaggaggaggagccaggacaggagggaagtgggagggagggtcccagcagaggactccaaatttaggcagcaggcatatgg
gatgggatataaaggggctggagcactgagagctgtcagagatttctccaaccc **gtcgac**acacttagccgtgttctttgcactttctgcatgtcccccgtctggcctggctgtccccagtggcttccccagtgtgacatggtgcatctctgccttacag

gttgtgacagttggaagtgtc**atg**tacaacatgcggcgattaagtctttcacccaccttttcaatgggatttcatctgttagttactgtgagtctcttattttcccatgtggaccatgtaattgctgagacagaaatggaaggagaaggaaatgaaactggtg
aatgtactggatcatattactgtaagaaaggggtgattttgcccatttgggaaccccaagacccttcttttggggacaaaattgctagagctactgtgtattttgtggccatggtctacatgtttcttggagtctctatcatagctgatcggttcatgtcctctat
agaagtcatcacatctcaagaaaaagaaataaccataaagaaacccaatggagagaccaccaagacaactgtgaggatctggaatgaaacagtttctaacctgaccttgatggccctgggatcttctgctcctgagattctcctttcagtaattgaagtg
tgtggccataacttcactgcaggagacctcggtcctagcaccatcgtgggaagtgctgcattcaatatgttcatcattattgcactctgtgtttatgtggtgcctgacggagagacaaggaagattaagcatttgcgtgtcttctttgtgacagcagcctggag
catctttgcctacacctggctttacattattttgtctgtcatatctcctggtgttgtggaggtctgggaaggtttgcttactttcttcttctttcccatctgtgttgtgttcgcttgggtagcggataggagacttctgttttacaagtatgtctacaagaggtatcga
gctggcaagcagagggggatgattattgaacatgaaggagacaggccatcttctaagactgaaattgaaatggacgggaaagtggtcaattctcatgttgaaaatttcttagatggtgctctggttctggaggtggatgagagggaccaagatgatgaag
aagctaggcgagaaatggctaggattctgaaggaacttaagcagaagcatccagataaagaaatagagcaattaatagaattagctaactaccaagtcctaagtcagcagcaaaaaagtagagcattttatcgcattcaagctactcgcctcatgactg
gagctggcaacattttaaagaggcatgcagctgaccaagcaaggaaggctgtcagcatgcacgaggtcaacactgaagtgactgaaaatgaccctgttagtaagatcttctttgaacaagggacatatcagtgtctggagaactgtggtactgtggccct
taccattatccgcagaggtggtgatttgactaacactgtgtttgttgacttcagaacagaggatggcacagcaaatgctgggtctgattatgaatttactgaaggaactgtggtgtttaagcctggtgatacccagaaggaaatcagagtgggtatcataga
tgatgatatctttgaggaggatgaaaatttccttgtgcatctcagcaatgtcaaagtatcttctgaagcttcagaagatggcatactggaagccaatcatgtttctacacttgcttgcctcggatctccctccactgccactgtaactatttttgatgatgacca
cgcaggcatttttacttttgaggaacctgtgactcatgtgagtgagagcattggcatcatggaggtgaaagtattgagaacatctggagctcgaggaaatgttatcgttccatataaaaccatcgaagggactgccagaggtggaggggaggattttgagg
acacttgtggagagctcgaattccagaatgatgaaattgt

gtgagtggcccgctacctcttctggtggccgcctccctccttcctggcctcccggagctgcgccctttctcactggttctctcttctgccgttttccgta**ggatcc**

A. Transcription efficiency.
B. circularization efficiency.
In the construct sequence, CMV promoter is labelled in green, and the heart promoter is labelled in red. Intron sequences are in blue, and the circular RNAs are in black.
